# ROCK inhibition with Y-27632 reduces joint inflammation and damage in serum-induced arthritis model and decreases *in vitro* osteoclastogenesis in patients with early arthritis

**DOI:** 10.3389/fimmu.2022.858069

**Published:** 2022-08-11

**Authors:** Angela Rodríguez-Trillo, Carmen Pena, Samuel García, Eva Pérez-Pampín, Marina Rodríguez-López, Antonio Mera-Varela, Antonio González, Carmen Conde

**Affiliations:** ^1^ Laboratorio de Reumatología Experimental y Observacional y Servicio de Reumatología, Instituto de Investigación Sanitaria de Santiago (IDIS), Hospital Clínico Universitario de Santiago de Compostela (CHUS), Servizo Galego de Saúde (SERGAS), Santiago de Compostela, Spain; ^2^ Laboratorio de Reumatología y Enfermedades Inmunomediadas (IRIDIS), Instituto de Investigación Sanitaria Galicia Sur (IIS Galicia Sur), Hospital Álvaro Cunqueiro, Vigo, Spain; ^3^ Servicio de Reumatología, Instituto de Investigación Sanitaria de Santiago (IDIS), Hospital Clínico Universitario de Santiago de Compostela (CHUS), Servizo Galego de Saúde (SERGAS), Santiago de Compostela, Spain

**Keywords:** rheumatoid arthritis, bone erosion, osteoclast (OC), K/BxN serum-transfer arthritis model, ROCK, Y-27632

## Abstract

Rheumatoid arthritis (RA) is a common chronic inflammatory disease affecting primarily peripheral joints, which is only partially controlled with current treatments. RA leads to pain, disability, deformities, and life expectancy shortening. Its pathogenesis is complex involving multiple cell types and signaling pathways that we incompletely understand. One of the pathways we have elucidated starts with WNT5A signaling and contributes to the aggressive phenotype of the RA synoviocytes through RYK-RhoA/ROCK signaling. Now, we have explored the contribution of ROCK to arthritis *in vivo*, using the K/BxN serum-transfer arthritis model; and to osteoclastogenesis, using the arthritis model and cells from patients with inflammatory arthritis. The mice and cells were treated with the ROCK inhibitor Y-27632 that caused a significant improvement of arthritis and reduction of osteoclastogenesis. The improvement in mouse arthritis was observed in the clinical evaluation and, histologically, in synovial inflammation, cartilage damage, bone erosion, and the abundance of multinucleated TRAP+ cells. Expression of inflammatory mediators in the arthritic joints, as assessed by real-time PCR, was also significantly reduced. The effect on bone was confirmed with *in vitro* assays using bone marrow precursors of arthritic mice and peripheral blood monocytes of patients with inflammatory arthritis. These assays showed dramatically reduced osteoclastogenesis and bone resorption. Overall, our findings suggest that ROCK inhibition could be part of a therapeutic strategy for RA by its dual action on inflammation and bone erosion.

## Introduction

Rheumatoid arthritis (RA) is an autoimmune disease characterized by chronic inflammation of peripheral joints that can lead to joint damage and irreversible disability. About 30% of patients have extra-articular manifestations as vasculitis, pericarditis, and interstitial lung disease that contribute to decreased quality of life and premature mortality (1, 2). Besides, RA has a notable socio-economic impact due to the high direct costs of patient care and the even higher indirect costs related to loss of working capacity. In recent decades, the development of biologic disease-modifying anti-rheumatoid drugs (DMARDs) blocking inflammatory cytokines, their downstream signaling pathways, or immune cells has much improved the prognosis of RA. However, many patients do not respond or partially respond to any DMARD. Moreover, many patients that initially responded to a given DMARD lose response over time (2–5). Therefore, it is still necessary to develop new treatment approaches.

A potential area for new RA drugs is the action over other RA players beyond the immune mediators and cells, which are only a fraction of the complex pathogenesis of RA. Two such cell types that are not targeted by current drugs but are critical in RA pathogenesis are the fibroblast-like synovial (FLS) cells and the osteoclasts. The two cells contribute to joint damage and deformities resulting in long-term sequelae, whereas the FLS also contribute to inflammation and disease persistence by adopting a characteristic aggressive phenotype (6–10). We have recently reported the involvement of non-canonical WNT5A in the aggressive phenotype of RA FLS. Specifically, WNT5A promoted the increased migration and invasiveness of RA FLS. These actions were mediated through the RYK-RhoA/ROCK signaling pathway (11). In this pathway, RYK is the cell membrane receptor specific for the non-canonical WNT, which after binding WNT5A induces activation of RhoA, a Rho family small GTPase, which in turn, leads to Rho-Kinase (ROCK) activation as one of the branches of the planar cell polarity (PCP) pathway (11). We considered particularly notable that treatment with ROCK inhibitor, Y-27632, reduced the activation of p38, ERK MAPK, and PI3K/AKT pathways and completely inhibited the WNT5A induced migration of RA FLS (11). These results are consistent with the known effects of ROCK and showed potential for drug targeting. In addition, there are several pieces of evidence indicating that the RhoA/ROCK pathway affects cartilage degradation (12, 13), bone resorption and formation (14–16), although none of these results were obtained in the context of inflammatory arthritis. In this regard, it is well known that osteoclasts are the pivotal mediators of bone resorption and that osteoclasts maturation and activation are mediated by macrophage colony-stimulating factor (M-CSF) and the receptor activator of NF-κB ligand (RANKL). M-CSF’s role is to enables survival, proliferation and recruitment of osteoclasts precursors through interaction with its receptor, c-Fms. The binding of RANKL with its receptor RANK, in turn, triggers osteoclast differentiation (17). This process is initiated by RANK-RANKL interaction, which leads to the recruitment of the TNF receptor-associated factor 6 (TRAF6) and the subsequent activation of the MAPK, AP-1 (c-Fos and c-Jun) and NFκB signaling pathways among others (18). Also, signaling mediated by TRAF cooperates with ITAM-mediated signals to upregulated the transcription of NFATc1, which is pivotal for the expression of osteoclast-specific genes (19). RANKL stimulation also activates signaling mediated by Rho, which is involved in several stages of osteoclastogenesis as the fusion of mononuclear cells to multinucleated osteoclasts, actin ring formation, podosome organization as well as migration and polarization of osteoclasts (14–16, 20).

ROCK proteins, ROCK1 and ROCK2, belong to the AGC superfamily of serine/threonine kinases and have their main activity in the actin-myosin cytoskeletal organization downstream of Rho activation (16, 21). Upon activation, ROCK phosphorylates more than thirty downstream substrates that are involved in subcellular processes as generation of actin-myosin contractile force, formation of stress fibers and focal adhesions, and in cellular functions as cell differentiation, changes in cell morphology, cell motility and migration, adhesion, cytokinesis and proliferation, and apoptosis and its crosstalk with autophagy (16, 21, 22). Besides, deregulation of the RhoA/ROCK pathway has been implicated in several pathological processes as cancer cell migration, angiogenesis, fibrosis (16, 21, 23, 24) and cardiovascular, renal and neurodegenerative disorders (25).

Here, we have analyzed the contribution of ROCK signaling to a murine model of arthritis resembling RA, the K/BxN serum transfer arthritis model. In this model, the transfer of arthritogenic antibodies induces a rapid and highly reproducible flare of arthritis, which shows the hallmarks of RA in the affected joints (26). We used Y-27632, one of the most widely used ROCK inhibitors. Y-27632 is specific for ROCK1 and ROCK2 with similar potency because it blocks access of ATP to the ATP-binding pocket on the kinase domain, which is the domain with the highest homology (92%) between the two ROCK isoforms (17). Our results showed that Y-27632 treatment improves the clinical severity of arthritis and reduces cartilage degradation and bone erosion. We have also found that Y-27632 reduces osteoclastogenesis and bone resorption in cells from arthritic mice. These effects may be relevant to the human disease because we observed a decrease in osteoclastogenesis and bone resorption by Y-27632 treatment in cells from patients with inflammatory arthritis.

## Material and methods

### Subjects and mice

Initial experiments were conducted using a mouse model of arthritis, subsequent comprobations were done in blood cells from patients with early arthritis. The KRN T cell receptor transgenic mice were a kind gift from C. Benoist and D. Mathis (Harvard Medical School, Boston, MA, USA). NOD/LTJ and C57BL/6J mice were purchased from Charles River Laboratories (Barcelona, Spain). K/BxN mice were generated by crossing KRN with NOD mice. Mice were maintained in the Center for Experimental Biomedicine (CEBEGA) from the University of Santiago de Compostela (USC). Animal experiments were done according to the Spanish regulations on the protection of animals used for experimental purposes (Real Decreto 1386/2018) and were approved by the Ethics Committee for Animal Research of the University of Santiago de Compostela and Galician authorities (15010/14/004 and 15012/2020/009).

Peripheral blood was obtained from nine patients with inflammatory arthritis in the visit before starting the first DMARD treatment. The patient’s characteristics are summarized in **Table 1**. The study was performed according to the Declaration of Helsinki, and it was approved by the Santiago-Lugo Ethics Committee for Research. All patients provided written informed consent.

**Table 1:** Characteristics of patients.

Parameters	Patients
Age (Mean ± SD)	60.7 ± 15.5
Gender Male	22.3%
Female	77.7%
RA patients	77.7%
Disease duration (weeks) NSAIDs Glucocorticoids ACPA POS	2.28 (1.03) 43% 100% 57.1%
NEG	42.8%
RF POS	71,4%
NEG	28.6%

Disease duration is presented as the mean (SEM) and represents time (weeks) between diagnosis date and sample collection date. NSAIDs, non-steroidal anti-inflammatory drugs; ACPA, Anti-citrullinated protein antibodies; RF, Rheumatoid factor.

### K/BxN serum-transfer arthritis

K/BxN serum was collected from 4-8 week-old arthritic K/BxN mice, pooled, and stored at -80°C until use (26). Arthritis was induced in 8 week-old C57BL/6J mice (18 females and 11 males) by intraperitoneal injection (i.p.) of 100 µl of K/BxN serum on days 0 and 2 (27, 28). Arthritic mice were treated with daily i.p. injections of 10 mg/Kg of Y-27632 (Selleckchem, Munich, Germany) or the same volume of physiological serum, as vehicle, from day 0 (1 hour before injection of 100 µl of K/BxN serum), until their sacrifice, day 10.

Arthritis was assessed every day at two levels in each of the four limbs by two observers using a semiquantitative clinical score. For ankle, tarsal, wrist and carpal joints (0: no swelling, 1: slight swelling and erythema, 2: moderate swelling and erythema, 3: severe swelling and erythema, and 4: maximal inflammation with joint rigidity). For finger and toe joints (0: no swelling, 1: swelling and erythema in 1 or 2 fingers, 2: swelling and erythema in 3 or 4 fingers, 3: swelling and erythema in 5 or 6 fingers, and 4: swelling and erythema in 7 or more fingers). The maximum possible score was 20 per mouse.

### Histological analysis of the mouse model

Histological analysis was performed as previously described (28, 29). Right hind limbs were prepared for histological analysis by dissecting the skin and sectioning the ankle joints. Specimens were fixed in 10% formaldehyde for 6 hours, demineralized in PBS 0.5M EDTA for 21 days, and embedded in paraffin. Sections were stained with hematoxylin and eosin (H&E) to assess inflammation and bone erosion. Ankle sections were stained with toluidine blue for analysis of cartilage damage. They were also stained for tartrate-resistant acid phosphatase (TRAP) to determine osteoclast activity using the TRAP/ALP stain kit (FUJIFILM Wako Chemicals Europe, Neuss, Germany) following the manufacturer’s indications.

Synovial inflammation was scored following a 0-4 scale: 0: no inflammation, 1: slight thickening of the synovial cell layer and some inflammatory cells in the sublining, 2: thickening of the synovial lining and moderate infiltration of the sublining, 3: more important thickening of the synovial lining and marked infiltration and 4: severe thickening of the synovial lining and severe infiltration.

Cartilage damage was evaluated following a 0-4 scale: 0: normal cartilage, 1: loss of proteoglycans, 2: proteoglycan loss and superficial cartilage erosion, 3: cartilage destruction reaching the central zone, and 4: marked cartilage destruction, with fissures extending near the subchondral bone.

Bone erosion was scored on a 0-4 scale: 0: normal bone, 1: a few small areas of resorption, 2: moderate areas of resorption, 3: frequent areas of resorption without structure alteration, and 4: full-thickness resorption areas with structure alteration. All scores were performed blind to mice group.

To analyze the inflammatory infiltrate, joint sections were deparaffined and stained with anti-F4/80 and anti-Ly6G antibodies (Abcam, Cambridge, UK). Three microphotographs of inflammatory infiltrate from each mouse were taken at 20x magnification with an Axio Vert.A1 microscope and analyzed using the Image J system.

### Real-time PCR analysis in mouse samples

Total RNA was obtained from wrist and ankle joints or cultured cells using the Speedtools total RNA extraction kit (Biotools, Madrid, Spain). Quantitative real-time PCR was performed in duplicate wells using SYBR Green qPCR master mix (Bimake, Munich, Germany) in a RotorGen thermocycler (Corbett, ThermoFisher Scientific, MA, EEUU). Gene expression was quantified by the comparative threshold cycle (Ct) method, using as normalization control the β-actin or Gadph genes. Primers were designed with the Primer3 Input Web version 4.1.0 and synthetized by Thermo Fisher Scientific (**Table 2**).

**Table 2:** Primers used for quantitative PCR.

Gene	Forward	Reverse
*Calcr*	CTCCAGTTCTTCAGGCTCCTAC	GTTGCAGTACAGACCTTCTCCT
*Ccl2*	ATCCCAATGAGTAGGCTGGA	TCTGGACCCATTCCTTCTTG
*c-Fms*	AACATATGGACTTCGCCCTC	CTAGCACTGTGAGAACCCCA
*c-Fos*	CCGCATGGAGTGTGTTGTTCC	GACCACCTCGACAATGCATGA
*Ctsk*	GCAGTATAACAGCAAGGTGGATG	CACTTCTTCACTGGTCATGTCTC
*Cxcl1*	GCTGGGATTCACCTCAAGAA	GTGGCTATGACTTCGGTTTG
*Cxcl5*	AGTGCCCTACGGTGGAAGT	TGCATTCCGCTTAGCTTTCT
*Dc-Stamp*	GGGCACCAGTATTTTCCTGA	CAGAACGGCCAGAAGAATGA
*Il1β*	GACCTTCCAGGATGAGGACA	AGCTCATATGGGTCCGACAG
*Il6*	GTTCTCTGGGAAATCGTGGA	TTCTGCAAGTGCATCATCGT
*Mmp3*	TTGTCCCGTTTCCATCTCTC	TGGTGATGTCTCAGGTTCCA
*Mmp9*	CATTCGCGTGGATAAGGAGT	TCACACGCCAGAAGAATTTG
*Mmp13*	TGATGAAACCTGGACAAGCA	TCATGGGCAGCAACAATAAA
*Nfact*	GGTCTTCCGAGTTCACATCC	GCCTTCTCCACGAAAATGAC
*Nos2*	CACCTTGGAAGAGGAGCAAC	AAGGCCAAACACAGCATACC
*Tnfα*	ACGTGGAACTGGCAGAAGAG	CTGATGAGAGGGAGGCCATT
*Actin*	GCTACAGCTTCACCACCACA	ATGCCACAGGATTCCATACC
*Gapdh*	AACTTTGGCATTGTGGAAGG	GGATGCAGGGATGATGTTCT

### Osteoclastogenesis and bone resorption *in vitro* assays for the mouse model

Bone marrow cells from tibiae and femurs of 8-week-old C57BL/6 arthritic mice were used to obtain osteoclasts as previously described (28). Once isolated, bone marrow cells were cultured in α-Minimum Essential Medium (α-MEM), 1% penicillin-streptomycin, 1% L-glutamine (all from Lonza, Barcelona, Spain) containing 10% v/v FBS (HyClone, Thermo Fisher Scientific). After 24h, non-adherent cells were cultured in 24-well plates at 2 x 10^6^ cells/well for TRAP staining and in 24-well plates coated with carbonate apatite (Cosmo Bio Co, LTD, Japan) for assessment of bone resorption activity. In both cases, cell differentiation was done in α-MEM, 10% FBS, 1% L-glutamine, 1% penicillin-streptomycin with 30 ng/mL MCSF (Peprotech EC, Ltd, London, UK) for 2 days. After, osteoclasts precursors were cultured for 8 days in complete α-MEM containing 40 ng/mL MCSF, 100 ng/mL RANKL (Peprotech) with 20, 40, 80 or 100 µM iROCK inhibitor, Y-27632 or vehicle, 0.04% DMSO. The differentiated osteoclasts were assessed by TRAP staining. In brief, osteoclasts were counted as the TRAP-positive cells with 3 or more nuclei in 20 images/well using an Axio Vert.A1 microscope (Zeiss, Oberkochen, Germany) at 40x magnification. In turn, the resorption was assessed as pit area at 10 days, after washing the wells with 5% sodium hypochlorite for 5 min. Pit area quantification was done using the Image J system in 10 images/well taken at 20x magnification.

### Bone matrix mineralization assay

Primary osteoblasts were obtained from tibiae and femurs of C57BL/6 arthritic mice and bone matrix mineralization assay was performed as previously described (28). Briefly, bones were removed and the epiphyses were cut into small pieces and cultured in osteogenic medium, α-MEM supplemented with 10% v/v FBS, 1% penicillin-streptomycin, 1% L-glutamine, and 0.28mM of ascorbic acid (Sigma Aldrich, Sant Louis, MO, EEUU). When the cells reached about 80% confluence, they were collected by trypsinization and cultured in 24-well plates at 15 x 10^4^ cells/well in an osteogenic medium containing 40, 80, or 100 µM Y-27632 or vehicle. After 10 days of culture, matrix mineralization analysis was done by von Kossa staining. Briefly, cells were fixed in 4% paraformaldehyde (Sigma Aldrich), stained with 5% silver nitrate solution under UV light for 1 h, and incubated in 1% pyrogallol (Sigma Aldrich) for 5min. Ten microphotographs/well were taken at 10x magnification with an Axio Vert.A1 microscope and analyzed using the Image J system.

### Osteoclast differentiation and bone resorption assays on monocytes from patients with inflammatory arthritis

Human osteoclasts differentiation was performed as previously described (30, 31). Peripheral blood mononuclear cells (PBMCs) were obtained by density gradient-centrifugation using Lymphoprep (ProGen Biotechnik Gmbh, Heidelberg, Germany). Monocytes were separated from PBMCs using a Percoll (Sigma Aldrich) gradient. The purified cells were cultured in 24-well plates at 2.5 x 10^5^ cells/well for 1 h in α-MEM supplemented with 1% FBS. After removing the non-adherent cells by washing, the adherent cells were cultured in α-MEM supplemented with 10% FBS, 1% penicillin-streptomycin, 1% L-glutamine, and 25 ng/mL de MCSF for 4 days. At this moment, cells were cultured in complete medium containing 25 ng/mL MCSF, 100 ng/mL RANKL and 20, 40, 80 or 100 µM Y-27632, or vehicle for 17 days. Half of the culture medium was renewed 3 times/week. Differentiated osteoclasts were assessed by or TRAP staining or real-time PCR. The TRAP-positive cells with 3 or more nuclei were counted in 20 images/well at 20x magnification using an Axio Vert.A1 microscope and the Image J analysis system. Also, osteoclasts were differentiated, following the protocol described above, in 24-well plates coated with carbonate apatite (Cosmo Bio) to analyze resorption activity. After 10 days of culture in complete medium containing 25 ng/ml MCSF, 100 ng/mL RANKL and 40, 80 or 100 µM Y-27632, or vehicle, wells were washed with 5% sodium hypochlorite for 5min and resorption pits were counted in 10 microphotographs/well at 20x magnification using the same system as above.

### Cell viability assay

Osteoclast precursors or primary osteoblasts were cultured in triplicates for each condition at an initial density of 2 × 10^4^ cells per well of 96-well plates. After 24h, the cells were treated with increasing concentrations of Y-27632 or vehicle, in 0,04% DMSO, in complete α-MEM containing 40 ng/mL MCSF and 100 ng/mL RANKL for osteoclasts or in complete α-MEM supplemented with 0.28mM of ascorbic acid, for osteoblasts. Cell viability was measured at 8 days for osteoclasts and 10 days for osteoblasts using a CellTiter-Glo luminescent cell viability assay (Promega Biotech Ibérica SL, Madrid, España) according to the manufacturer’s instructions.

### Statistical Analysis

Differences between the experimental groups were assessed with the Wilcoxon matched-pairs test or the Mann Whitney U test using the GraphPad Prism software Prism 9.0 (GraphPad Software, San Diego, CA, USA). The analysis of ROCK inhibitor in the arthritis model was assessed by ANOVA repeated measures, using the statistical program IBM SPSS (IBM SPSS Statistics, Hampshire, UK). P values < 0.05 were considered significant.

## Results

### ROCK inhibition with Y-27632 reduces the severity of serum-transfer arthritis

**Figure 1:**
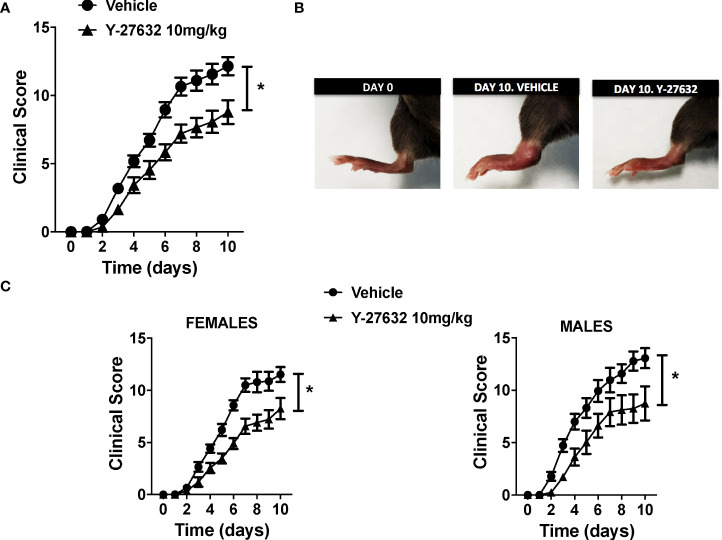
ROCK inhibition with Y-27632 reduced the severity of serum-transfer arthritis in mice. **(A)** Clinical arthritis score in C57BL/6J mice receiving 100 µl of K/BxN serum on days 0 and 2 and treated every day with 10 mg/Kg of Y-27632 (n = 14) or vehicle (n = 15). **(B)** Representative pictures of a mouse rear paw at day 0, day 10 after arthritis induction and treated with vehicle, and day 10 after arthritis induction and treated with Y-27632. **(C)** Clinical arthritis scores in 18 C57BL/6 females and 11 males after treatment with Y-27632 or vehicle. Values are the Mean ± Standard error of the mean (SEM). *p < 0.05 (ANOVA repeated-measures test). Y-27632: ROCK inhibitor.

To ascertain the role of ROCK, we compared serum-transfer arthritis induced in mice injected with the inhibitor Y-27632 or physiological serum. The group of mice treated with Y-27632 included 14 C57BL/6J mice (8 females and 6 males) that received 10 mg/Kg of the inhibitor daily from day 0 to 10 in the arthritis course. The control group consisted of 15 C57BL/6J mice (10 females and 5 males) injected with physiological serum. Two blinded observers evaluated the severity of arthritis with a clinical score assessing redness, swelling, and motility of the ankle, tarsal, wrist, carpal, finger, and toe joints up to a maximum of 20 points. The assessment was done from day 1 until day 10, a time when the maximum clinical score is reached for the K/BxN serum-transfer arthritis in our animal facility. The incidence of arthritis was 100% in the two groups of mice: treated with Y-27632 and control. Also, the time course of the disease was similar (**Figure 1A**). However, the severity of arthritis was attenuated in the mice treated with Y-27632 compared with the control mice (p < 0.0001, **Figures 1A, B**). At the end of the study, the mean score in the control mice was 12.14 ± 0.67, whereas in the mice treated with Y-27632 was 8.78 ± 0.87. The reduction in severity was not gender-dependent as a similar attenuation was observed in females and males (**Figure 1C**).

### Decreased synovial inflammation, cartilage damage and bone erosion in arthritic mice treated with Y-27632

**Figure 2:**
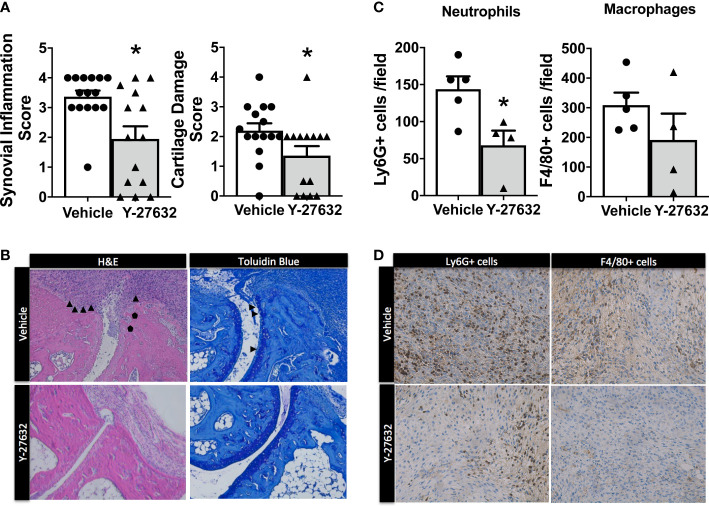
Decreased synovial inflammation and cartilage damage in arthritic mice treated with Y-27632 . **(A)** Comparison of the histologic scores of synovial inflammation and cartilage damage in ankle and forefoot joints of arthritic mice treated with either Y-27632 (n = 14) or vehicle (n = 15). **(B)** Representative histologic images of the joints stained with hematoxylin and eosin (H&E) and toluidine blue. ▲ indicates synovitis and invading pannus and ● indicates subchondral bone erosion in the H&E sections and ▲ indicates proteoglycan loss (destaining of superficial cartilage) and erosion of superficial cartilage in the Toluidin blue stained sections. Values are the Mean ± Standard error of the mean (SEM). *p < 0.05 (Mann-Whitney test). **(C)** Number of cell Ly6G+ neutrophils or F4/80 macrophages in the inflammatory infiltrate of joints from arthritic mice treated with Y-27632 (n = 5) or vehicle (n = 4), with a clinical score of 2-3. **(D)** Representative images of the joint infiltrate stained with the anti-Ly6G+ or anti-F4/80 antibodies. Values are the Mean ± Standard error of the mean (SEM). *p < 0.05 (Mann-Whitney test).

We assessed the histological changes in the tibiotalar and forefoot joints ten days after arthritis induction. The analysis revealed a significant decrease in synovial inflammation and cartilage damage in the mice treated with Y-27632 relative to the controls (**Figures 2A, B**). In effect, the mice injected with Y-27632 showed a 42.3% lower synovial inflammation than controls (p = 0.015). A similar reduction, 38.6%, was observed in cartilage damage (p = 0.028). We also investigated the impact of Y-27632 treatment on the synovial infiltration by neutrophils and macrophages, pivotal cells in the K/BxN serum transfer arthritis (32, 33), using immunohistology. Joint tissue from mice treated with Y-27632 or vehicle and a clinical score of 2-3 were stained with anti-neutrophils (anti-Ly6G) or anti-monocytes/macrophages (anti-F4/80) specific antibodies. These analyses showed that Y-27632 treatment reduced by 47.3% neutrophils (p = 0.016) and by 38.2% macrophages (non-significant) infiltration (**Figures 2C, D**).

**Figure 3:**
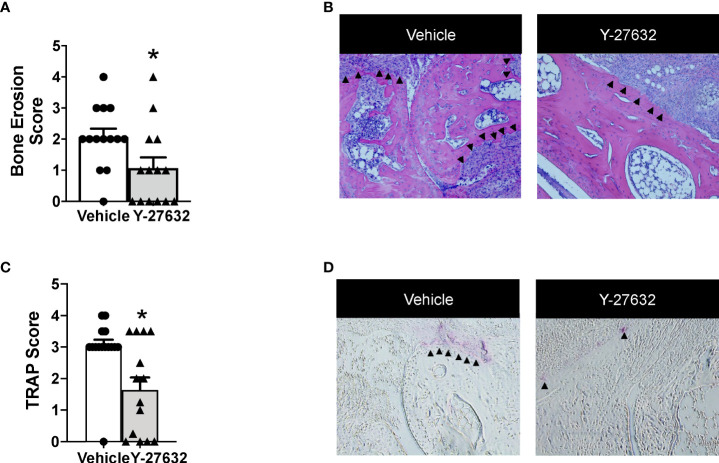
Treatment with Y-27632 decreased bone erosion in arthritic mice. Comparison of the histologic score of bone erosion **(A)** and tartrate-resistant acid phosphatase (TRAP) activity score **(C)** in the joints of the same mice shown in Figure 2. Representative images of bone erosion **(B)** and TRAP activity **(D)** assessment. ▲indicates invasive pannus tissue penetrating into subchondral bone areas **(B)** and TRAP + bone resorbing osteoclasts **(D)** in the two groups of mice. Values are the Mean ± SEM. *p < 0.05 (Mann-Whitney test).

Bone erosion was also highly reduced after treatment with Y-27632 (**Figures 3A, B**). In effect, the score was a 48.3% lower in mice treated with Y-27632 than that observed in controls (p = 0.021). Bone erosion was also highly reduced after treatment with Y-27632 (**Figures 3A, B**). In effect, the score was a 48.3% lower in mice treated with Y-27632 than in controls (p= 0.021). To further assess the influence of Y-27632 treatment on inflammatory osteoclastogenesis, we analyzed the TRAP activity in the joint sections. As shown in **Figures 3C, D** there was a marked reduction of multinucleated TRAP+ osteoclasts in the mice treated with Y-27632 compared with controls (p = 0.029).

### ROCK inhibition with Y-27632 diminished the expression of inflammatory mediators and metalloproteinases

**Figure 4:**
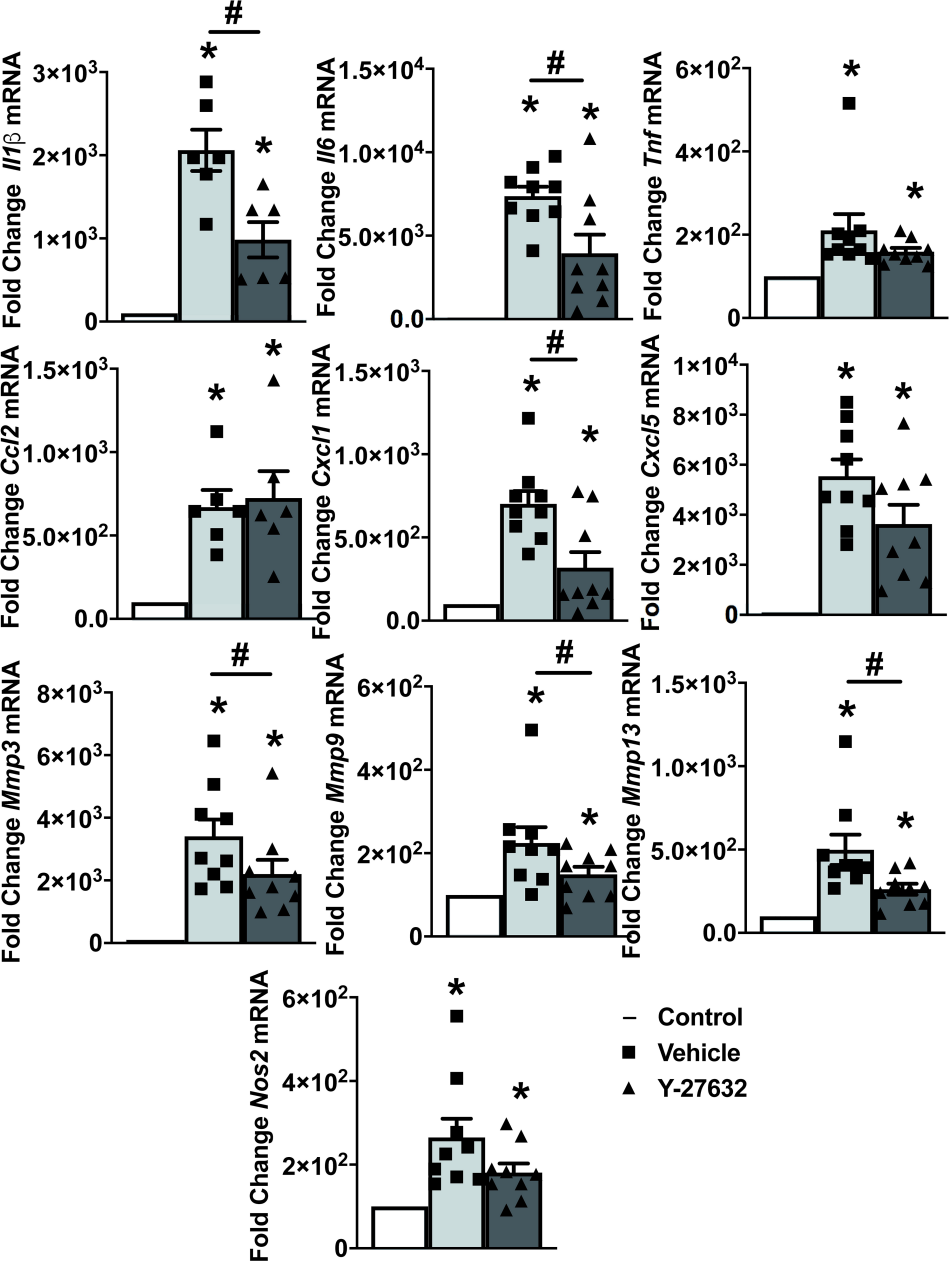
ROCK inhibition with Y-27632 diminished the expression of inflammatory mediators and metalloproteinases. Comparison of cytokines (Il-1β, Il-6, TNFα), chemokines (Ccl2, Cxcl1, Cxcl5), metalloproteinases (Mmp3, Mmp9, Mmp13), and Nos2 mRNA expression in the joints of non-arthritic control mice and arthritic mice treated with Y-27632 or vehicle. Values represent Mean ± SEM from 6-9 mice per group. *p < 0.05 for the comparison with the non-arthritic control mice and # < 0.05 for comparison between the two groups of arthritic mice (Mann-Whitney test).

To explore the mechanisms underlying the decrease in arthritis severity, we analyzed the expression of ten inflammatory mediators and metalloproteinases in the joint tissues from 9 Y-27632-treated and 9 vehicle-treated mice, on the 10^th^ day of the arthritis course (**Figure 4**). Also, we analyzed the expression in the joints of mice without arthritis as a reference. The results showed on one side the changes in mRNA expression induced by arthritis and on the other, the effect of Y-27632. Regarding arthritis, all the assessed genes were increased in the arthritic mice (**Figure 4**). Treatment with Y-27632 significantly reduced the overexpression induced by arthritis except in the case of *Tnfα, Cxcl5, Nos2* and *Ccl2*. The first three genes did not show a statistically significant regulation by Y-27632, although showed a trend to a more limited overexpression, although it was non-significant (p = 0.09; p = 0.13; p = 0.07, respectively). *Ccl2*, in contrast, was not modulated at all by Y-27632 despite being overexpressed in arthritic mice (**Figure 4**).

### Y-27632 prevents osteoclast differentiation and bone resorption without changes in viability

**Figure 5:**
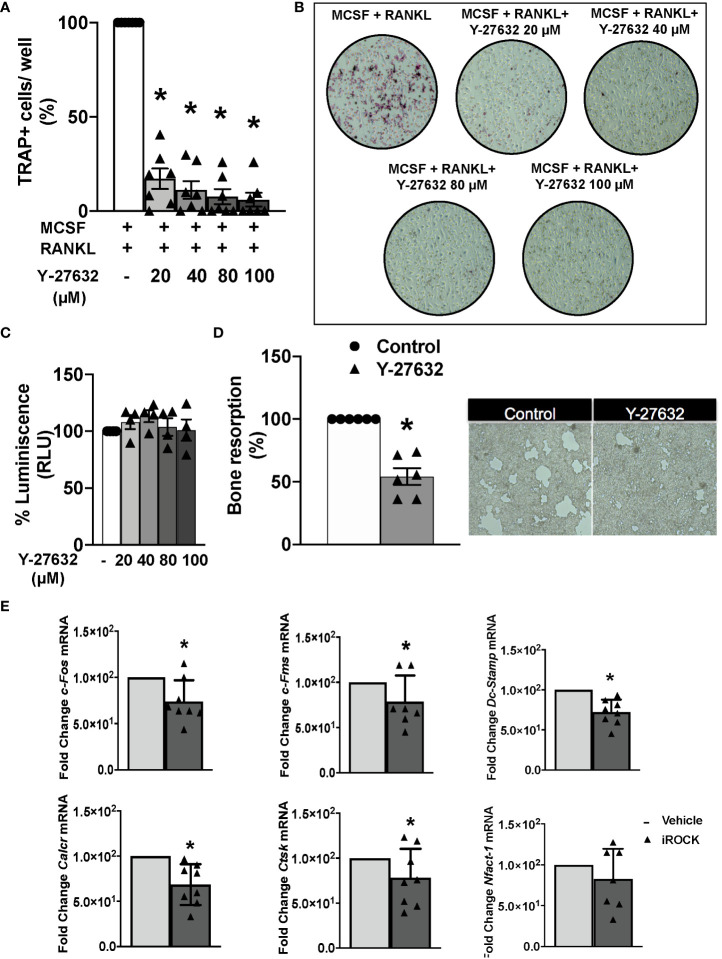
Inhibition of ROCK with Y-27632 prevents osteoclast differentiation and bone resorption in mice. **(A)** Percentage of TRAP+ multinucleated cells differentiated from bone marrow (BM) precursors of arthritic mice in the presence of MCSF and RANKL for 8 days and the indicated concentrations of the ROCK inhibitor Y-27632. Positive control was considered 100%. **(B)** Representative images of TRAP+ cells. **(C)** Cell viability of BM precursors treated for 8 days with the indicated concentrations, determined using the Cell Titer-Glo luminescent assay kit. Obtained luminescence for positive control was considered 100%. **(D)** Carbonate apatite resorption area produced by differentiated osteoclasts and representative images. Resorption produced by control group was considered 100%. **(E)** Gene expression of c-Fos, c-Fms, Dc-Stamp, Calcr, Ctsk and Nfatc-1 in BM precursors differentiated to osteoclasts following the same protocol as in A and quantified by real-time PCR. Values are the mean ± SEM of cells from 7 arthritic mice in **(A)**, 4 in **(C)**, 6 in **(D)** and 7-8 in **(E)**. *p < 0.05 (Wilcoxon test).

We wanted to explore the Y-27632 effect on bone erosion that was revealed by the histological analysis. Therefore, we assayed the differentiation and function of osteoclasts derived from arthritic mice bone marrow precursors. The differentiation was done by incubation with MCSF and RANKL for 8 days. In the differentiation medium, we added vehicle or different concentrations of Y-27632. All the Y-27632 concentrations, from 20 to 100 µM, achieved a highly significant decrease in the number of multinucleated TRAP+ cells compared to the obtained with vehicle (**Figures 5A, B**). This reduction was dose-dependent, with the frequency of TRAP+ cells ranging from 20 to 6% of the vehicle, corresponding to the lowest and highest doses of Y-27632, respectively (**Figures 5A, B**). This remarkable effect was not attributable to cell damage induced by the ROCK inhibitor because no decrease in cell viability was observed in precursors treated with any dose of Y-27632 (**Figure 5C**).

Next, we determined the effect of 40µM Y-27632 on the osteoclast bone resorption function. The bone marrow precursors were differentiated in wells covered with carbonate-apatite. After 10 days, we quantified the area of the resorption pits appearing on the plates. There was a 46% reduction of the resorption pit area in the wells treated with Y-27632 compared to the vehicle control (**Figure 5D**). We also assessed the effect of Y-27632 on the expression of osteoclastogenic markers in the cells differentiated in these conditions (**Figure 5E**). Treatment with Y-27632 reduced the expression of all the analyzed markers in comparison with vehicle (**Figure 5E**). The decrease was significant for the two markers of osteoclast activity (calcitonin receptor, *Calcr*, and cathepsin K, *Ctsk*), the transcription factor *c-Fos* and the Mcsf receptor, *cfms*, that contribute to osteoclast differentiation, and *Dc-Stamp*, that is involved in osteoclast fusion and differentiation. The decrease of the transcription factor *Nfatc1*, were also reduced after treatment, although the decrease was not-significant (**Figure 5E**).

### Y-27632 did not modulate osteoblast matrix mineralization

**Figure 6:**
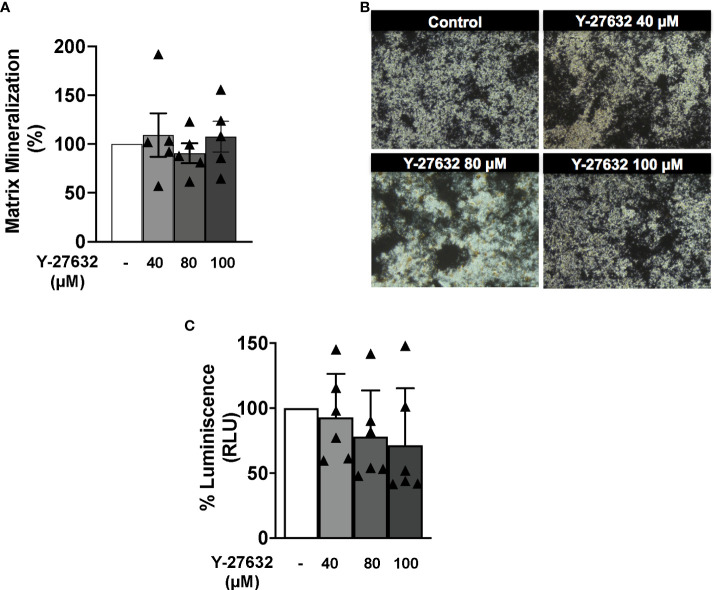
The ROCK inhibitor Y-27632 did not modulate osteoblast activity. **(A)** Percentage of matrix mineralization produced by *in vitro* differentiated osteoblasts from arthritic mice, treated with different concentrations of Y-27632. Control without treatment was considered 100%. **(B)** Representative imagens are shown. **(C)** Cell viability in the differentiation of osteoblasts treated for 10 days with the indicated Y-27632 concentrations. Viability was determined using the Cell Titer-Glo luminescent assay kit and expressed relative to the cultures without Y-27632 (100%). Values are the mean ± SEM of cells from 5 arthritic mice in **(A)** and 6 in **(C)**.

We analyzed the effect of Y-27632 on osteoblasts derived from arthritic mice. These experiments were done with primary osteoblasts obtained from the epiphysis of tibiae and femurs of arthritic mice. Our results showed no differences in osteoblast mineralization function between ROCK inhibition and control (**Figures 6A, B**). The lack of effect was shown by quantifying the mineralized nodules with the von Kossa staining. The ROCK inhibitor did not modify mineralization at any tested dose. In addition, we observe that Y-27632 did not affect cell viability in these experiments (**Figure 6C**).

### Inhibition of ROCK with Y-27632 also reduced osteoclast differentiation and bone resorption in cells from patients with inflammatory arthritis

Given the effect of Y-27632 on osteoclastogenesis in arthritic mice, we wanted to know whether this treatment also modulates osteoclastogenesis in patients with arthritis. Therefore, we obtained PBMCs of nine patients with early inflammatory arthritis that have not yet received any DMARD to analyze osteoclast differentiation and function. Seven patients were subsequently classified as having RA. Other characteristics of the patients are shown in **Table 1**.

**Figure 7:**
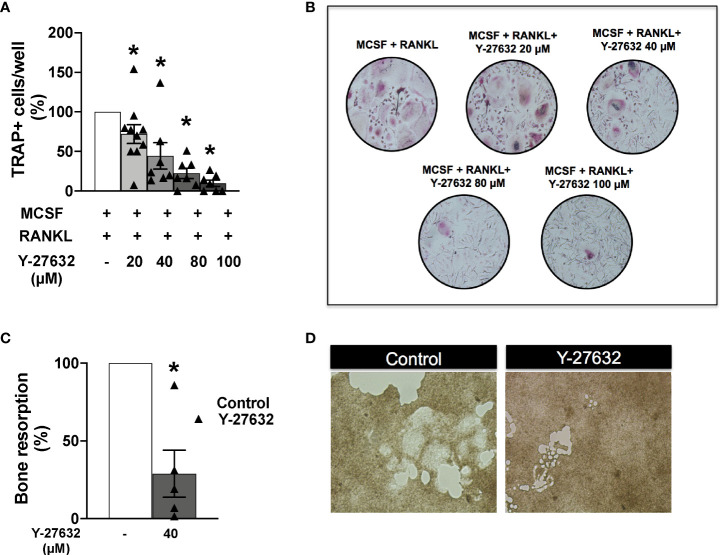
Inhibition of ROCK with Y-27632 also reduced osteoclast differentiation and bone resorption in cells from patients with inflammatory arthritis. **(A)** Percentage of TRAP+ multinucleated cells differentiated from blood monocytes of patients with inflammatory arthritis in the presence of MCSF and RANKL and the indicated concentrations of Y-27632. **(B)** Representative images of experiments in **(A)**. **(C)** Bone resorption area produced by differentiated osteoclasts from patients with inflammatory arthritis treated with 40µM Y-27632 relative to the same cells without Y-27632. **(D)** Representative pictures of the resorption pits in **(C)** Positive control (MCSF+RANKL) was considered 100%. Values are the Mean ± SEM of cells from 5-6 patients with inflammatory arthritis. *p < 0.05 (Wilcoxon test).

Monocytes obtained from the patients’ PBMCs were differentiated to osteoclasts by incubation with MCSF and RANKL for 21 days. Several concentrations of the ROCK inhibitor or the vehicle were added to the differentiation medium as described in the Material and Methods section. Assessment of the number of differentiated TRAP+ osteoclasts showed a similar result to the obtained in mice: all doses of Y-27632 significantly reduced osteoclast differentiation relative to the control. This reduction was dose-dependent (**Figures 7A, B**), showing the maximum decrease, to 10% of the control, with the 100 µM dose of Y-27632.

We also analyzed the bone resorption ability of osteoclasts differentiated in the presence of Y-27632. We used the same assay as for mouse cells: quantification of the resorption pits in carbonate-apatite plates by osteoclasts differentiated in the presence of 40 µM of Y-27632. As shown in **Figures 7C, D** there was a reduction of 72% in the resorption pit areas compared with the vehicle control, confirming the downmodulation of bone resorption by ROCK inhibition.

## Discussion

Our results have revealed that ROCK inhibition with Y-27632, can interfere with osteoclastogenesis and bone resorption in inflammatory arthritis. The results supporting this role include the histological analysis of joints from the Y-27632 treated arthritic mice and the *in vitro* assays done with mouse and human cells. The *in vitro* assays showed decreased osteoclastogenesis and less ability of differentiated osteoclasts to bone resorption, both in the human and the mouse models. Y-27632 treatment also improved other lesions in the arthritic mice, cartilage damage and synovial inflammation, characterized by a decrease of the infiltrating neutrophils and, less clearly, of macrophages. Besides, we have been the first to analyze the effect of Y-27632 on the expression of inflammatory mediators in the mice arthritic joints. We observed a decrease in important players in inflammation as *Il-1β*, *Il-6*, *Cxcl1*, and in several metalloproteases, *Mmp-3*, *Mmp-9*, and *Mmp-13*.

No previous analysis of the ROCK role in arthritis had addressed osteoclastogenesis or bone erosion. Here, we have discovered a marked reduction of the bone erosions and the number of TRAP+ cells in arthritic mice treated with Y-27632. The *in vitro* assays confirmed these findings showing a direct interference with osteoclastogenesis and a decrease in the osteoclastogenic markers analyzed representing early and late stages of osteoclast differentiation. These results excluded that the reduction of bone erosions was a mere consequence of the attenuation of inflammation resulting from the treatment with Y-27632. In effect, bone erosions in arthritis result from the combination of multiple signals that include high levels of RANKL in FLS, B, and T cells (34); the inflammatory cytokines TNFα, IL-1, and IL-6, both in RANKL dependent and independent pathways (34–36); and anti-CCP and other RA autoantibodies (37). The *in vitro* assays performed in this work have other remarkable aspects. One is that they included osteoclastogenesis induced by MCSF and RANKL treatment of bone marrow cells and peripheral blood monocytes, which are the two cell populations most likely to contribute osteoclast precursors for bone erosions in RA (38). These experiments try to mimic focal bone erosion in arthritis that is mediated by osteoclasts located in the interphase between inflammatory synovial tissue and periarticular bone. Osteoclast’s precursors in the synovial pannus derive from monocytes-lineage cells of the bone marrow, which migrate into the joints after passing through secondary lymphatic organs (8). *In vivo*, the osteoclast precursors are exposed to RANKL and M-CSF produced by neighboring cells. Specifically, RANKL is produced by osteoblasts, osteocytes, dendritic cells, T cells, and in arthritis, also by fibroblast-like synoviocytes (FLS), whereas M-CSF is secreted by osteoblasts and osteocytes. Another remarkable point is the sensitivity of osteoclastogenesis to the lowest assayed Y-27632 doses. Whereas it is well known the role of RhoA in osteoclastogenesis (14), the knowledge about the role of ROCK is very scarce. In effect, RhoA is known to participate in the migration of preosteoclast and mature osteoclast, podosome rearrangement, osteoclast polarization, formation of the sealing zone, and bone resorption (14, 39–42). However, there was no known role of ROCK in osteoclastogenesis until recently, when Nakata *et al.* reported enhanced *in vitro* osteoclast differentiation after ROCK inhibition with Y-27632 and HA-1077 (15). This result is at odds with our results, with the already discussed roles of RhoA in osteoclast differentiation and function (14, 39–42), and with the known involvement of ROCK in migration, invasion, and formation of filopodia or uropods in other cells (43–45). However, there is also previous evidence of the complex spatiotemporal interplay between different Rho GTPases that could justify in some way the contrasting *in vitro* results of Nakata et al. (15). On the contrary, the consistency of our *in vitro* and *in vivo* results and the compatibility with the known roles of RhoA increase confidence in the generality of our findings.

The involvement of ROCK in other aspects of arthritis beyond osteoclastogenesis was already known by the experiments with cells from RA patients and treatment of arthritic rats (11, 46, 47). They include our previous work where we have found the RYK-RhoA/ROCK signaling pathway is critical for the Wnt5a-enhanced migration and invasiveness of RA FLS (11). The experiments using rats have shown that inhibition of ROCK ameliorates multiple features of arthritis: synovial hyperplasia, inflammatory cell infiltration, pannus formation, and cartilage erosion (46, 47). The two studies used different arthritis models, CIA that involves T and B cells, and AIA that involves T cells, respectively (46, 47). Our current experiments have extended the generality of the beneficial effects because our study is on a different model and in mice. This extension is meaningful because the K/BxN serum transfer model does not involve T or B cells (48, 49). Therefore, our results mean that attenuation of arthritis can be the consequence of ROCK inhibition on the innate immune component of the disease. In effect, we observed a reduction of the clinical score, synovial inflammation, and cartilage damage comparable to the previously reported in the CIA and AIA models. The previous studies identified ROCK signaling is needed for TNFα and IL-1β-induced activation of NF-κB in FLS and other cells from RA patients (46, 47); for IL-1β-induced production of IL-6, IL-8, and GM-CSF by FLS, and of IL-6 and IL-8 by endothelial cells, and for the increased adhesion of neutrophils and endothelial cells from RA patients (46). All these effects could contribute to the beneficial effect of ROCK inhibition on arthritis, but none requires T or B cells. We think it is necessary to address the question of the possible beneficial effects for arthritis by ROCK inhibition on these cells.

We have also found that treatment of mice with Y-27632 significantly downmodulated of most overexpressed inflammatory mediators in joints. Ccl2, which was upregulated in arthritis, did not show downmodulation after ROCK inhibition. This Ccl2 result is similar to the reported in an IL-1β- or TNFα-stimulated epithelial cell line, in which treatment with Y-27632 did not affect Ccl2 expression but downmodulated another chemokine, Cxcl8 (50). Therefore, it seems that the Ccl2 regulation shows ROCK independence. Regarding the downmodulation of the other soluble inflammatory mediators and the previously reported inhibition of NF-κB, it seems very likely they contribute to the attenuation of arthritis.

Another aspect of our results worth mentioning is the absence of changes in the bone mineralization assay. We performed this assay with primary osteoblasts from the arthritic mice, which are characteristics to consider because osteogenesis is very context-dependent (51–55). Specifically, osteogenesis depends on the differentiation of the osteoblast precursors, mechanical cues from the environment, and the inflammatory milieu, among other factors. The two first factors are known to determine the involvement of the RhoA/ROCK pathway in osteogenesis (51, 53–55). However, no study has yet addressed if inflammation affects the participation of RhoA/ROCK in osteogenesis. Regarding the differentiation of the osteoblast precursors, RhoA/ROCK pathway activation promotes osteogenesis in mesenchymal stem cells (51–53), but inhibits osteogenesis in a variety of cells. The latter include cell lines like MC3T3-E1 (15, 56), C2C12 (57), and Saos-2 (58), but also the murine neonatal calvarial cells (43 54). These cells represent more advanced stages of osteoblast differentiation. Therefore, a complex regulatory scenario has been hypothesized requiring RhoA/ROCK signaling for mesenchymal stem cell commitment and differentiation to osteoblasts, but its suppression for the terminal differentiation (54, 59).

Overall, our work has confirmed the attenuation of experimental arthritis by Y-27632 treatment, including the new aspect of a specific decrease of osteoclastogenesis without a negative impact on osteogenesis. A strength of the study is the demonstration of decreased osteoclastogenesis *in vivo*, in arthritic mice, and *in vitro*, in mice and humans. However, a limitation of our study is the possibility of inhibition of other kinases beyond ROCK1 and ROCK2. In effect, Y-27632 is considered as a selective ROCK inhibitor, although at high doses it can also inhibit other kinases (60–62). We expect that future development of more specific ROCK inhibitors will permit to avoid this limitation for the understanding of ROCK role in inflammatory arthritis.

Another question that would require a different experimental setting is the effect of ROCK inhibition for treatment, once arthritis is established, in place of prevention, as done here. The rapid course of arthritis in the serum transfer model prevents treatment before extensive joint damage and bone erosions have appeared. In effect, by 10 days the affected joints are extensively damaged and inflammation subsides thereafter. Inflammation can be maintained by repeated serum transfers, but there is no evidence of additional joint destruction (63–65).

It is conceivable that ROCK inhibition could become part of a future therapeutical strategy for RA. This perspective will resemble an already tested approach to include denosumab, an anti-RANKL monoclonal antibody, in the management of RA patients (66, 67). Currently, most patients are treated with Disease-Modifying Anti-Rheumatic Drugs (DMARD), either as monotherapy or as combination therapy (68). These drugs control inflammation and stop the erosive process in most patients, but not in all. A reason for the persistent erosions seems to be residual joint inflammation, despite clinical remission, as demonstrated with Magnetic Resonance Imaging (69–71). Therefore, the combination of denosumab with DMARDs was tested in clinical trials that demonstrated a significant improvement in bone erosions (66, 67).

In the present work, we suggest that ROCK inhibition could play a role in RA treatment, as a drug to add to DMARD in patients at particular risk of erosions.

## Data availability statement

The raw data supporting the conclusions of this article will be made available by the authors, without undue reservation.

## Ethics statement 

The studies involving human participants were reviewed and approved by Santiago-Lugo Ethics Committee for Research. The patients/participants provided their written informed consent to participate in this study. The animal study was reviewed and approved by Ethics Committee for Animal Research of the University of Santiago de Compostela and Galician authorities.

## Author contributions

A R-T performed experiments and participated in the analysis of data and contributed to writing the manuscript. CP performed experiments. EP-P, MR-L, AM-V provided patients or clinical and laboratory data and revised the manuscript. SG and AG participated in the analysis of data and contributes to writing the manuscript. CC designed research, managed the project, analyzed data and wrote the manuscript. All authors have read and approved the manuscript.

## Funding

Instituto de Salud Carlos III, PI20/01266, PI17/01660 and Redes Temáticas de Investigación Cooperativa en Salud (RETICS) Program, RD16/0012/0014, co-funded by the European Union.

## Acknowledgments

The authors thank the patients for their contributions.

## Conflict of interest

The authors declare that the research was conducted in the absence of any commercial or financial relationships that could be construed as a potential conflict of interest.

## Publisher’s note

All claims expressed in this article are solely those of the authors and do not necessarily represent those of their affiliated organizations, or those of the publisher, the editors and the reviewers. Any product that may be evaluated in this article, or claim that may be made by its manufacturer, is not guaranteed or endorsed by the publisher.
